# Effects of different negative pressure cupping interventions on inflammatory response and motor function recovery in delayed onset muscle soreness

**DOI:** 10.3389/fspor.2025.1622688

**Published:** 2025-08-25

**Authors:** Xu Song, Teng Ma, XianYou Cui

**Affiliations:** ^1^ School of Physical Education, Zhejiang Guangsha Vocational and Technical University of Construction, Dongyang, China; ^2^ Department of Physical Education, Xinjiang Second Medical College, Xinjiang, China

**Keywords:** delayed onset muscle soreness, pain, negative pressure cupping therapy, pressure value

## Abstract

**Introduction:**

This study examined the impacts of different negative pressure cupping therapies (*N*PCT) on pain relief, functional recovery, and inflammatory regulation in delayed onset muscle soreness (DOMS) after high-intensity exercise, with the aim of clarifying the dose-effect relationship.

**Methods:**

In this study, 55 healthy male participants aged 18–25 were selected and divided into 5 groups: the control group (CTR; *n* = 11) and NPCT groups at different levels (−25 kPa, −35 kPa, −45 kPa, and −55 kPa; *n* = 11 in each group). A high-intensity protocol, which included 6 sets of lunges, squats, and squat jumps, was adopted to induce DOMS in the quadriceps femoris. Immediately after the exercise, the DGN-6 vacuum device was used for a 10 min NPCT treatment. The research outcomes included visual analog scale (VAS) pain scores, lower extremity explosive strength tests (30-meter sprint and standing long jump), joint range of motion (ROM), and serum biomarkers [CK, LDH, and inflammatory cytokines (IL-6, TNF-α, and Hsp27)]. These were assessed at the baseline and 24 h after the intervention.

**Results:**

NPCT groups exhibited significantly lower VAS scores than the CTR group (−55 kPa: 1.57 ± 0.79 vs. 6.14 ± 0.69; *P* < 0.05), and the efficacy was pressure-dependent (−55 kPa > −4 kPa > −35 kPa; *P* < 0.01). Functional recovery was significantly improved in NPCT groups (30-meter sprint: 0.27 s; standing long jump: 0.08 m; *P* < 0.01). Knee ROM increased by 5.71° at −55 kPa and 6.43° at −45 kPa (*P* < 0.05). Biochemically, CK/LDH levels normalized in −45 kPa and −55 kPa groups (*P* < 0.05). Meanwhile, the levels of IL-6 and TNF-α decreased significantly (*P* < 0.05), and these changes were correlated with Hsp27 expression (*r* = 0.42–0.49; *P* < 0.05).

**Discussion:**

These findings demonstrate that NPCT at pressures ranging from −45 kPa to −55 kPa is most effective in alleviating DOMS by enhancing hemodynamics and modulating the anti-inflammatory response, which supports its integration into post-exercise rehabilitation protocols.

**Clinical Trial Registration:**

https://www.chictr.org.cn/showprojEN.html?proj=263241, Chinese Clinical Trial Registry (ChiCTR) (Registration NO.: ChiCTR- 2500098071, 03/03/2025).

## 1 Introduction

Delayed onset muscle soreness (DOMS) arises from exercise-induced ultrastructural damage to skeletal muscle (1). It is characterized by localized inflammatory responses and transient functional impairments resulting from microtears in muscle fibers. As one of the most prevalent exercise-related injuries, DOMS typically exhibits clinical features like mechanical hyperalgesia (dull pain and tenderness), muscle stiffness, localized swelling, reduced muscle strength, and altered joint kinematics (2–4). These pathological changes can diminish explosive power, sprinting ability, and jumping height, and disrupt muscle recruitment patterns during eccentric exercise. Consequently, the reduced tolerance of the muscle-tendon complex to mechanical loads increases the risk of secondary injuries (5).

Negative pressure cupping therapies (NPCT) are traditional Chinese medical therapies that promote wound healing and accelerate the recovery of exercise-related injuries by improving local microcirculation (6). Through suction on specific body surfaces, NPCT can raise pain thresholds, relieve fatigue, and reduce perceived pain intensity (7–9). Mechanistically, NPCT can put local tissues in a state of high oxygen availability and low metabolic demand, enhance blood flow, raise tissue temperature, and accelerate metabolic turnover (10). The mechanical pressure generated during cupping stimulates auto-hemolysis, promotes inflammation resolution, and modulates systemic physiological states (11).

The therapeutic effects of NPCT can be ascribed to two main mechanisms: (1) Hemodynamic theory: Negative pressure can improve local microcirculation, accelerate the clearance of metabolic byproducts (e.g., lactate), enhance anti-inflammatory capacity, and promote muscle repair (9, 12, 13); (2) Gate control theory of pain: Mechanical stimulation can activate cutaneous mechanoreceptors, whose neural impulses propagate faster than nociceptive signals. This triggers neuromodulation at the spinal level, elevates pain thresholds, and produces analgesia. Supplementary mechanisms include reduced muscle tension and improved blood perfusion via negative-pressure-applied lifting and kneading actions (14, 15).

Although NPCT are widely used for post-exercise pain management, systematic research on its pressure-dependent effects on DOMS remains limited. Current evidence suggests that negative pressure ranging from −20 kPa to −50 kPa can relieve muscle pain; however, its effects on inflammatory markers [e.g., interleukin-6 (IL-6) and tumor necrosis factor-α (TNF-α)] and muscle injury indicators [e.g., creatine kinase (CK) and lactate dehydrogenase (LDH)] lack robust validation (16). This study aims to fill these gaps by evaluating the efficacy of NPCT in DOMS recovery under different pressures (−25 kPa to −55 kPa), elucidating its mechanism of action, and providing references for personalized rehabilitation protocols.

## 2 Materials and methods

### 2.1 Experimental approach to the problem

This study enrolled healthy male university students as participants. The decision to include only male participants was based on existing evidence indicating that gender differences can influence post-exercise serum CK concentration (17). Under the same exercise load, females have significantly lower CK activity than males. This approach aimed to minimize gender-related confounding effects. The inclusion criteria for the participants were as follows: (1) Aged 18–25 years; (2) No history of metabolic, hormonal, orthopedic, cardiovascular, or infectious diseases, and no use of chronic medications; (3) During the study period, apart from daily activities like walking and climbing stairs, structured exercise such as running and weightlifting must be avoided; (4) Full-time students.

Table 1 shows the demographic characteristics of the participants, with no significant differences in baseline age, height, or weight among the study groups.

**Table 1:** Basic characteristics of the study participants.

Variable	CTR (*n* = 11)	−25 KPa (*n* = 11)	−35 KPa (*n* = 11)	−45 KPa (*n* = 11)	−55 KPa (*n* = 11)
Age/(year)	22.00 ± 2.58	22.14 ± 1.21	22.43 ± 1.62	22.00 ± 1.63	22.43 ± 1.99
Height/(cm)	1.76 ± 0.05	1.74 ± 0.04	1.72 ± 0.02	1.80 ± 0.08	1.76 ± 0.04
Mass/(kg)	75.14 ± 5.49	69.29 ± 6.29	73.86 ± 5.21	70.00 ± 6.38	73.71 ± 4.64

### 2.2 Participants

*A priori* power analysis was conducted using G*Power 3.1 (18) to determine the minimum sample size required to detect intergroup differences. Based on previous studies on cupping therapy and muscle recovery (19, 20), the analysis parameters were set as follows: the significance level (α) was set at 0.05, the statistical power (1-β) was set at 0.8, and the effect size (Cohen's f) was set at 0.5. The analysis results indicated that for the one-way analysis of variance (ANOVA) model, each group needed at least 10 participants (degrees of freedom = 4 and total sample size ≥ 50).

**Figure 1:**
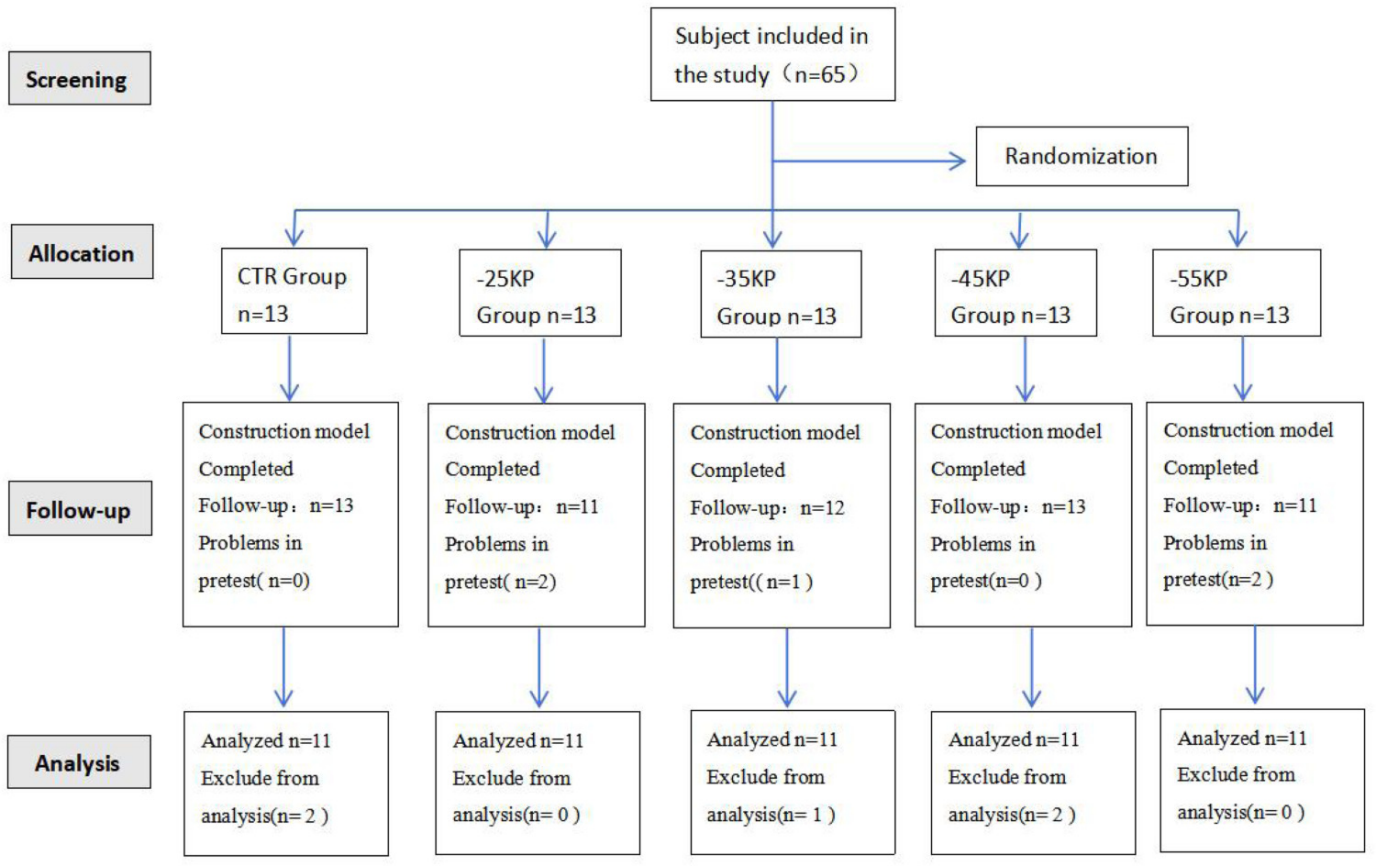
Experimental grouping diagram.

To account for potential confounding factors (e.g., inter-individual metabolic variability and compliance fluctuations) and reserve a 15% buffer for data loss or incomplete data, the total sample size was increased to 65 participants (5 groups, 13 participants per group). Out of the 65 participants, a total of 10 were excluded from the analysis. The reasons for exclusion were as follows: 5 participants were unable to persevere throughout the intervention period, 2 had personal schedule conflicts, and 3 had data loss. Consequently, only 55 participants were included in the final analysis. Participants were randomly assigned to 5 groups (control, −25 kPa, −35 kPa, −45 kPa, and −55 kPa) using a computer-generated block randomization method implemented with SPSS 22.0 software (block size = 5). Prior to the start of the intervention, the allocation sequence was concealed in sequentially numbered opaque sealed envelopes. The randomization process is shown in Figure 1.

The study protocol was approved by the Academic Research Ethics Committee of Karamay Central Hospital (Approval No.: YL–2024–97). Before participating in the study, all participants read and signed informed consent forms. This study was conducted in accordance with the Declaration of Helsinki, approved by the Institutional Review Board of the Academic Research Ethics Committee of Karamay Central Hospital (Ethics Committee No.: YL–2024-97), and registered in the Chinese Clinical Trial Registry (ChiCTR) (Registration NO.:ChiCTR–2500098071, 03/03/2025).

### 2.3 Procedures

#### 2.3.1 Delayed onset muscle soreness induction protocol

A DOMS model was established using a high-intensity interval training (HIIT) protocol adapted from the work of Hung et al. This HIIT exercise regimen, which has been proven to induce DOMS in the quadriceps muscle (21), consists of the following exercises:

1. Lunges: 6 sets × 20 repetitions, with 30-second intra-set rest and 3-minute inter-set rest.
2. Squats: 6 sets × 20 repetitions, with 30-second intra-set rest and 3-minute inter-set rest.
3. Squat jumps: 6 sets × 20 repetitions, with 30-second intra-set rest and 3-minute inter-set rest.

Warm-up procedure: Before starting the exercise, participants were required to complete a standardized 10-minute warm-up. Five minutes at a speed of 8 km/h on a motorized treadmill (Model XT-2000, TechnoGym). Dynamic stretching: Five minutes targeting quadriceps and hamstrings (e.g., leg swings and walking lunges).

During the DOMS modeling process, participants had to continue until one of the following termination criteria was reached: (1) Muscle fatigue prevented further exercise (unable to complete the action even with verbal encouragement); (2) The self-reported VAS pain score ≥ 8.This dual-criterion approach ensured participant safety while effectively inducing DOMS.

The validation criteria for the DOMS model were as follows: At 24 h after exercise, the VAS and objective indicators were employed to assess DOMS symptoms, including localized muscle soreness, palpable muscle stiffness, thigh circumference (TC), joint range of motion (ROM), and body biochemical indicators.

#### 2.3.2 NPCT protocol

Medical-grade plastic cups (outer diameter: 60 mm) were applied to the quadriceps femoris under varying negative pressures (−25 kPa, −35 kPa, −45 kPa, and −55 kPa). The operational steps were as follows: Participants were positioned in a supine position with their lower limbs relaxed. Placed 4–6 cups along the quadriceps muscle belly, with a 0–2 cm gap between cups, to ensure uniform pressure distribution. The DGN-6 multifunctional vacuum device (Kangjian Technology) was utilized to maintain a constant negative pressure for 10 min. Strict adherence to sterile operating procedures was necessary to prevent skin injury and ensure participant comfort.

#### 2.3.3 Subjective pain assessment

The VAS was used to assess DOMS pain both before and after exercise (22). It is a subjective measurement instrument accompanied by verbal descriptions, with a rating scale ranging from 0 (no pain) to 10 (extremely sore). This instrument demonstrates high reliability in assessing pain intensity, with an intragroup correlation coefficient of 0.97 (23). Participants were asked to rate their muscle soreness during active knee extension in an unloaded condition.

This scale was categorized as follows: 0 (no pain), 1–3 (mild pain, no sleep disturbance), 4–6 (moderate pain, mild sleep disturbance), and 7–10 (severe pain, significant sleep disruption). After performing 3 standardized squats (knee flexion angle: 90° ± 5°), participants marked their perceived pain levels on the VAS.

#### 2.3.4 Measurement indicators

Lower extremity explosive strength test: In studies on athletes' lower-limb explosive strength, tests like the 30-meter sprint test and the standing long jump test are frequently used as key indicators. Moreover, these tests have also been widely applied in studies involving other populations (24, 25).

Standing long jump test: Participants stood with their feet parallel to each other behind the starting line, keeping their knees naturally straight, and then jumped with maximum force. The horizontal distance from the heel to the starting line was measured using a tape measure (precision: 0.005 m; compliant with ISO 5725:1994), and the result was recorded to two decimal places.

30-meter sprint test: Participants assumed a standing starting position and sprinted at maximum speed upon receiving a signal (electronic whistle, WS-101). Timing started with the first movement and ended when the torso crossed the finish line. The timing was carried out using photoelectric gates (Brower Timing Systems) with a precision of 0.01 s.

##### 2.3.4.1 Body morphological measurements

TC: TC serves as a metric for detecting acute changes in thigh volume. An increase in the measurement suggests edema resulting from exercise-induced muscle damage (26). The reliability of measuring thigh muscle volume is extremely high (intragroup correlation coefficient = 1) (27). TC was measured at 5, 10, and 15 cm proximal to the upper edge of the patella using semi-permanent markers. Participants were placed in a supine position, with their lower limbs abducted at an angle of 15° and kept in a relaxed state. A NIST-certified tape measure (Lufkin W606PM) was placed perpendicular to the limb's longitudinal axis under a constant tension of 0.5 N ± 0.1 N. Each point was measured 3 times (precision: 0.1 cm).

ROM: Joint ROM represents the angle traversed during joint movement and is an indicator of joint mobility. It is also a key indicator for assessing the extent and degree of joint movement function impairment (28). The ROM measurement method followed a previous study (29), which evaluated joint ROM of the subjects' quadriceps femoris by measuring knee joint ROM and hip flexion ROM.

Knee ROM: To ensure consistency, the lateral epicondyle, greater trochanter, and lateral malleolus of the femur were marked with semi-permanent markers. The fulcrum of the goniometer was positioned on the lateral epicondyle of the femur, its fixed arm was aligned with the greater trochanter, and its moving arm was aligned with the lateral malleolus. The subject was laid supine on the massage table. The angle of the 3-point line was measured by the goniometer when the knee joint was extended, and this was the extension angle of the test leg. Then, the angle between the 3 points was measured when the test leg's knee joint was maximally flexed, and this was used as the flexion angle of the test leg. Knee ROM was calculated as the difference between the knee extension and flexion angles.

Hip flexion ROM: To ensure consistency, the lateral epicondyle, the midline, and the greater trochanter of the femur were marked with semi-permanent markers. The fulcrum of the goniometer was placed above the greater trochanter of the tested leg. The moving arm was aligned with the midline of the femur, with the lateral epicondyle of the femur serving as a reference point. The participant's posterior ankle was gently lifted while keeping the knee joint fully extended. The participant was instructed not to lift their pelvis off the massage table throughout the entire movement. The participant was then asked to raise the straight leg as high as possible until reaching the maximum stretch point.

The results from 3 trials for each measurement were averaged.

Blood collection and processing: After high-intensity eccentric exercise or unfamiliar types of exercise, the concentrations of muscle damage markers [CK, LDH, IL-6, TNF-α, and heat shock protein 27 (Hsp27)] in the blood will increase. For guidance on these test indicators, relevant studies on DOMS using methods such as vibration training, massage, and drug therapy can be considered (30–33).

Venous blood was collected between 08:00 and 10:00. The participants were required to keep their torso perpendicular to the ground and their arms steadily placed on the platform. The participants were instructed to refrain from moderate-to-vigorous physical activity [>3 metabolic equivalents (METs)] within 24 h before sampling. Whole blood was aliquoted into 5 ml vacuum coagulation tubes (BD Biosciences). The tubes were gently inverted 8 times, by 180° each time, and then allowed to coagulate at room temperature for 30 min. The serum was separated by centrifugation at 3,000 × g for 10 min at 4°C. The serum was transferred to pre-cooled microcentrifuge tubes, divided into 5 equal aliquots, and stored at −80°C. The biomarkers of skeletal muscle injury (CK and LDH), inflammatory cytokines (IL-6, TNF-α, and Hsp27) were assayed using an Architect C-8,000 (Abbott Laboratories, USA) automated chemistry analyzer. All samples were processed to prevent repeated freeze-thaw cycles.

### 2.4 Statistical analysis

This study employed SPSS 22.0 software for statistical analysis and GraphPad Prism 9.0 software for data visualization. The results of each indicator were presented as the mean ± standard deviation (SD). Initially, the normality of the data was assessed using the one-sample Kolmogorov–Smirnov test. For intragroup comparison (before and 24 h after exercise), a paired-sample *t*-test was applied. For intergroup comparison, the significance level was set at *α* = 0.05. After confirming the normality (Shapiro–Wilk test) and homogeneity of variance (Levene's test) of the data, one-way ANOVA was used to analyze intergroup differences, followed by *post hoc* multiple comparisons using the least significant difference (LSD) method.

The Shapiro–Wilk test was used to assess the normality of the data, and Pearson correlation analysis was performed. According to Hopkins, a correlation coefficient (r) of 0 signifies no correlation, 0.1 indicates a low correlation, 0.3 indicates a moderate correlation, 0.5 indicates a high correlation, 0.7 indicates a very high correlation, and 0.9 indicates an extremely strong correlation (32). The analysis was conducted at a significance level of *α* < 0.05, with a 95% confidence interval (CI).

## 3 Results

### 3.1 Subjective visual pain indicators

**Figure 2:**
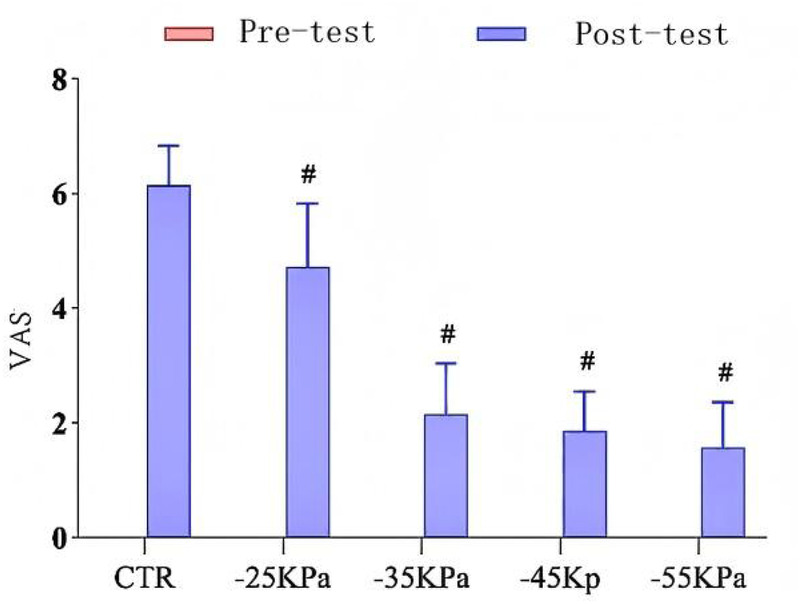
Effects of different levels of negative-pressure cupping on the VAS. # indicates a statistically significant difference when compared to the CTR group.

Figure 2 presents the VAS scores of each study group. At baseline (24 h before exercise), all participants reported no pain (VAS score: 0.00 ± 0.00), and there was no significant intergroup difference (*P* > 0.05). Twenty-four hours after inducing DOMS, the VAS scores of intervention groups were significantly lower than those of the control group (CTR: 6.14 ± 0.69; NPCT groups: 1.57 ± 0.79–4.71 ± 1.11; *P* < 0.05). Moreover, a pressure-dependent therapeutic efficacy was observed. Specifically, the −55 kPa group showed the greatest pain reduction (VAS score: 1.57 ± 0.79), followed by the −45 kPa group (1.86 ± 0.69), the −35 kPa group (2.14 ± 0.90), and the −25 kPa group (4.71 ± 1.11) (*P* < 0.05).

### 3.2 Indicators for lower extremity explosive strength test

**Figure 3:**
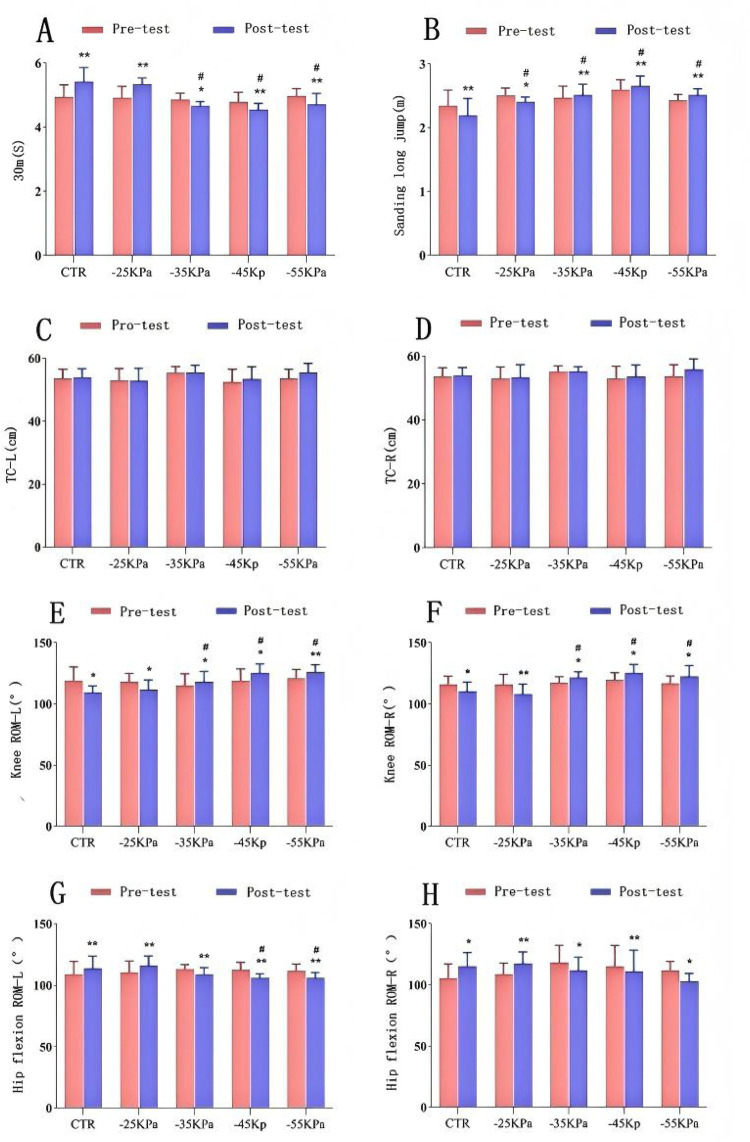
Effects of different negative-pressure cupping on: **(A)** 30-meter sprint; **(B)** standing long jump; **(C,D)** thigh circumference; **(E,F)** knee flexibility; **(H,G)** hip flexibility. Data presented as mean ± SD. Significant intragroup differences: *P < 0.05, **P < 0.01, ***P < 0.001. Significant intergroup differences: #*P* < 0.05.

Figures 3A,B illustrate the changes in the results of the 30-meter sprint and standing long jump. Intragroup comparisons (before and 24 h after exercise) revealed statistically significant differences in 30-meter sprint performance across all groups (*P* < 0.05). Specifically, the CTR group and the −25 kPa group exhibited significant declines in 30-meter sprint performance at 24 h after exercise (*P* < 0.01). In contrast, the 30-meter sprint performance of −55 kPa, −45 kPa, and −35 kPa groups recovered at 24 h after exercise (*P* < 0.01, *P* < 0.01, and *P* < 0.05, respectively), with improvements of 0.27 s, 0.24 s, and 0.2 s, respectively. Intergroup comparative analysis showed no statistically significant difference in 30-meter sprint performance across the groups 24 h before DOMS modeling (*P* > 0.05). However, at 24 h after modeling, −55 kPa, −45 kPa, and −35 kPa groups exhibited better recovery than the −25 kPa group and the CTR group, and there were statistically significant differences compared with the CTR group (*P* < 0.05). The −55 kPa group demonstrated the greatest recovery efficacy in exercise capacity, followed by the −45 kPa group and the −35 kPa group.

Intragroup comparisons (before and 24 h after exercise) revealed statistically significant differences in standing long jump performance across all groups (*P* < 0.05). Specifically, the CTR group and −25 kPa group exhibited significant declines in standing long jump performance at 24 h after exercise (*P* < 0.05). In contrast, the standing long jump performance of −55 kPa, −45 kPa, and −35 kPa groups recovered at 24 h after exercise (*P* < 0.01), with improvements of 0.08 m, 0.06 m, and 0.04 m, respectively. Intergroup comparative analysis showed no statistically significant difference in standing long jump performance across the groups 24 h before DOMS modeling (*P* > 0.05). However, 24 h after modeling, −55 kPa, −45 kPa, −35 kPa, and −25 kPa groups exhibited statistically significant differences compared with the CTR group (*P* < 0.05). Compared with the −25 kPa group and the CTR group, −55 kPa, −45 kPa, and −35 kPa groups demonstrated superior recovery. Among them, the −45 kPa group had the greatest recovery efficacy, with a performance improvement of 2.65 ± 0.16 s, followed by the −55 kPa group (2.51 ± 0.10 s) and the −35 kPa group (2.51 ± 0.17 s).

### 3.3 Body morphometric measurements

#### 3.3.1 Thigh circumference

The improvement effects on TC are depicted in Figures 3C,D. Within each group, there was no statistically significant difference between the pre-exercise and 24 h post-exercise measurements (*P* > 0.05). Additionally, there was no statistically significant difference in left or right TC between the intervention groups and the CTR group either 24 h before or after DOMS modeling *(P* > 0.05).

#### 3.3.2 Knee range of motion and hip flexion range of motion

Figures 3E–H illustrate the changes in knee ROM and hip flexion ROM 24 h before and after exercise. Paired-sample t-tests for intragroup comparisons revealed statistically significant differences in left and right knee ROM between the pre-exercise and 24 h post-exercise time points across all groups (*P* < 0.05). At 24 h after DOMS modeling, the CTR group and the −25 kPa group exhibited significant declines in knee ROM (*P* < 0.05). The CTR group had reductions of 9.28° (left) and 4.29° (right), while the −25 kPa group declined by 6.43° (left) and 7.85° (right). In contrast, −35 kPa, −45 kPa, and −55 kPa groups demonstrated significant improvements in knee ROM (*P* < 0.05). In the −35 kPa group, left knee ROM increased by 2.86° and right knee ROM increased by 4.29°. In the −45 kPa group, left knee ROM increased by 6.43° and right knee ROM increased by 5.71°. In the −55 kPa group, left knee ROM increased by 5.00° and right knee ROM increased by 5.71°. *Post hoc* multiple comparisons indicated that the CTR group had the poorest knee ROM recovery, while the −45 kPa group and the −55 kPa group exhibited the greatest improvement in knee ROM.

Paired-sample *t*-tests for intragroup comparisons also revealed statistically significant differences between the pre-exercise and 24 h post-exercise measurements across all groups in terms of hip flexion ROM (*P* < 0.05). At 24 h after DOMS modeling, the CTR group and the −25 kPa group exhibited significant declines in hip flexion ROM (*P* < 0.05). In the CTR group, left hip flexion ROM decreased by 5.00° and right hip flexion ROM decreased by 10.00°. In the −25 kPa group, left hip flexion ROM decreased by 5.71° and right hip flexion ROM decreased by 8.57°. In contrast, −35 kPa, −45 kPa, and −55 kPa groups demonstrated significant improvements in hip flexion ROM (*P* < 0.05). In the −35 kPa group, left hip flexion ROM increased by 4.29° and right hip flexion ROM increased by 6.43°. In the −45 kPa group, left hip flexion ROM increased by 6.43° and right hip flexion ROM increased by 4.29°. In the −55 kPa group, left hip flexion ROM increased by 5.72° and right hip flexion ROM increased by 8.57°. *Post hoc* multiple comparisons indicated that the CTR group had the poorest hip flexion ROM recovery, while −45 kPa and −55 kPa groups demonstrated the greatest improvements.

### 3.4 Body biochemical indicators

#### 3.4.1 CK and LDH

**Figure 4:**
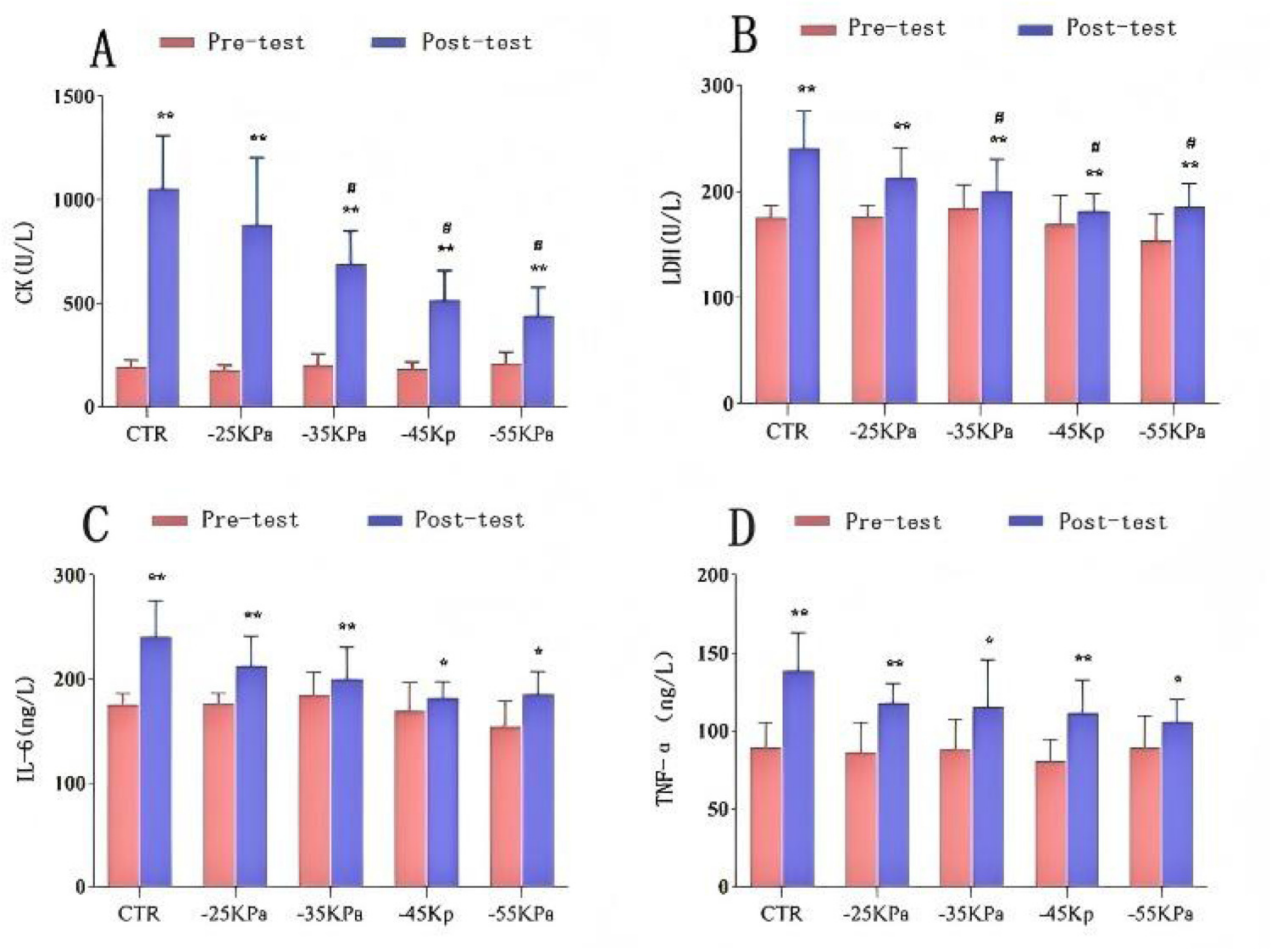
Effects of different pressure cupping on biochemical markers: **(A)** CK levels; **(B)** LDH levels; **(C)** IL-6 levels; **(D)** TNF-α levels. Data are presented as mean ± SD. Significant intragroup differences: *P < 0.05, **P < 0.01, ***P < 0.001. Significant intergroup differences: #*P* < 0.05.

Figures 4A,B depict the changes in serum CK and LDH levels among the participants. Paired-sample t-tests for intragroup comparisons revealed statistically significant differences in serum CK and LDH levels before and 24 h after exercise across all groups (*P* < 0.05). Notably, elevated serum CK and LDH levels were observed after exercise.

One-way ANOVA for intergroup comparisons showed no statistically significant differences in serum CK or LDH levels among groups 24 h before DOMS modeling (*P* > 0.05). However, significant intergroup differences were found 24 h after DOMS modeling (*P* < 0.05). *Post hoc* multiple comparisons demonstrated that the CTR group exhibited the highest increase in both serum CK and LDH levels. Notably, the −45 kPa group nearly restored serum LDH levels to the baseline (*P* < 0.05), while the −55 kPa group restored serum CK levels to pre-modeling values (*P* < 0.05).

#### 3.4.2 IL-6, TNF-α, and Hsp27

Figures 4C,D illustrate the changes in serum IL-6, TNF-α, and Hsp27 levels. Paired-sample *t*-tests for intragroup comparisons revealed statistically significant differences in serum IL-6 and TNF-α levels before and 24 h after exercise across all groups (*P* < 0.05). Notably, elevated serum IL-6, TNF-α, and Hsp27 levels were observed after exercise. The CTR group exhibited the greatest increase in TNF-α levels. One-way ANOVA for intergroup comparisons showed no statistically significant differences in serum IL-6 or TNF-α levels among groups 24 h before or after DOMS modeling (*P* > 0.05).

Similarly, intragroup comparisons demonstrated statistically significant increases in serum Hsp27 levels before and 24 h after exercise across all groups (*P* < 0.05). Intergroup comparisons revealed no statistically significant differences in serum Hsp27 levels among groups 24 h before or after DOMS modeling (*P* > 0.05).

Notably, as shown in Table 2, Pearson correlation analysis revealed significant positive relationships between Hsp27 and inflammatory markers: Hsp27 vs. IL-6: *r* = 0.420 (95% CI: 0.18–0.61), *P* = 0.012; Hsp27 vs. TNF-α: *r* = 0.494 (95% CI: 0.25–0.68), *P* = 0.003. Both correlations were classified as moderate (|*r*| = 0.30–0.50) according to Hopkins et al. (2,009) (34).

**Table 2:** Pearson correlation analysis of Hsp27 with IL-6 and TNF-α. Data are presented as correlation coefficients (*r*) along with their corresponding *P*-values. Statistical significance is defined as *P* < 0.05.

Variable Pair	r-value (*r*)	Classification	*P*-value (*p*)
Hsp27 vs. IL-6	0.420	Moderate	0.012
Hsp27 vs. TNF-α	0.494	Moderate	0.003

## 4 Discussion

Skeletal muscle serves as the primary driving force for human locomotion (35, 36). Exercise promotes health and enhances physical fitness. However, repetitive high-intensity or unaccustomed muscular activity can lead to micro-injuries in skeletal muscle fibers (1, 37). DOMS is a prevalent musculoskeletal symptom among athletes (38). Symptoms start to appear 6–12 h after exercise, peak within 24–72 h, and typically subside within 5–7 days (severe cases lasting ≤ 7 days) (38–40). DOMS can reduce joint ROM, muscle strength, and proprioception, thus affecting athletic performance and recovery efficiency (41–43).

Since the mid-1990s, NPCT has emerged as a non-pharmacological intervention that effectively relieves myofascial trigger points, reduces muscle pain, and improves muscle tension. Based on this, this study examined the therapeutic effects of NPCT on DOMS under different negative pressures, aiming to identify the optimal negative pressure parameters and elucidate the underlying mechanisms. This study deepens our understanding of NPCT and offers practical guidance for its application in post-exercise recovery in the general population.

Muscle pain is a subjective post-exercise sensation influenced by exercise load, intensity, duration, and participant age (44). The VAS is a safe, effective, and subjective assessment tool widely used to evaluate the severity of muscle pain (45) and is the most commonly used assessment method.

In this study, the VAS was utilized to assess the pain-alleviating effects of NPCT on DOMS under different negative pressures. When comparing the muscle pain levels between the CTR group and intervention groups (−25 kPa, −35 kPa, −45 kPa, and −55 kPa) 24 h after intervention, it was found that all intervention groups significantly mitigated the severity of DOMS and reduced muscle pain (*P* < 0.05). These interventions effectively relieved the subjective pain that occurs after high-intensity exercise. In contrast, the CTR group experienced more pronounced post-exercise pain and showed the greatest variability. The results demonstrated that interventions at −45 kPa and −55 kPa were the most effective in relieving muscle soreness, nearly restoring the pain to the pre-exercise baseline levels. This aligns with the results reported by Hammons (8) and Stephens (46), confirming that specific negative pressure levels are essential for NPCT to stimulate local tissues, enhance blood circulation, and clear metabolic byproducts, thereby reducing pain. A larger negative pressure can induce more significant localized hemodynamic changes (47), which accounts for the dose-dependent improvement in therapeutic efficacy as the negative pressure increases. These findings indicate that NPCT significantly alleviates DOMS-related pain and demonstrates substantial benefits in treating myofascial trigger points and muscle pain.

Lower limb explosive power is a core indicator for evaluating athletic performance (40), which can be measured through tests such as the standing long jump, countermovement jump (CMJ), vertical jump, 10-second sprint, and 30-meter sprint. Intragroup comparisons of 30-meter sprint and standing long jump performance revealed that −35 kPa, −45 kPa, and −55 kPa groups showed overall improvement, while the CTR group and the −25 kPa group showed declines. Intergroup comparisons demonstrated that −35 kPa, −45 kPa, and −55 kPa groups achieved optimal sprint performance 24 h after cupping (*P* < 0.05). Similarly, the standing long jump performance of these groups also improved, increasing by 0.04 m, 0.06 m, and 0.08 m, respectively, whereas the CTR group and the −25 kPa group declined. These results align with the research findings of Wolska Beata (48) and Sungyeon Oh (46), in which symptoms related to DOMS, such as muscle soreness, inflammation, stiffness, and swelling, can impair joint ROM, muscle strength, proprioception, and muscle function, hindering athletes and laborers from attaining optimal performance (49). The observed improvements may stem from enhanced blood circulation (50), reduced muscle adhesion, and improved elasticity, which may be mediated by activating the AMPK/mTOR signaling pathway, thus promoting muscle protein synthesis, energy metabolism, and accelerating post-exercise recovery.

In summary, NPCT, especially under higher negative pressures, can effectively compensate for the insufficient lower limb explosive power caused by DOMS and promote rapid recovery.

Eccentric exercise often causes soft tissue damage and swelling. A single bout of high-intensity exercise can lead to muscle congestion and ultrastructural damage, resulting in tissue fluid exudation and subsequent swelling. This study examined left and right TC before and 24 h after high-intensity exercise to explore the effects of different negative pressures on muscle swelling during DOMS recovery. The results showed that the muscle swelling caused by high-intensity exercise was similar among −25 kPa, −35 kPa, −45 kPa, and −55 kPa groups after cupping. However, no significant intragroup or intergroup differences were observed (*P* > 0.05), which may be attributed to secondary tissue damage caused by cupping negative pressure. Therefore, TC is not a reliable indicator for evaluating the efficacy of cupping in DOMS recovery.

Previous studies (51, 52) on DOMS have indicated that symptoms including muscle soreness, stiffness, inflammation, and swelling can lead to an increase in TC, a reduction in joint ROM, and these symptoms indirectly reflect muscle tissue swelling. Research examining the effects of NPCT on flexibility, tissue stiffness, and joint mobility has demonstrated significant improvements in flexibility (53), pain threshold (54), and muscle contraction capacity (55). However, Murray et al. (50) and systematic reviews (51, 56, 57) suggest that while cupping may transiently alter ROM, it fails to bring about lasting changes. Therefore, there is still inconsistency in the impact of cupping on joint mobility, and further research is needed.

In this study, at 24 h after exercise, quadriceps knee ROM decreased significantly. However, the intervention group exhibited improvement (*P* < 0.05). Notably, the groups with negative pressures ranging from −45 kPa to −55 kPa achieved the optimal recovery. In the −35 kPa group, left and right knee ROM increased by 2.86° and 4.29°, respectively. In the −45 kPa group, left and right knee ROM increased by 6.43° and 5.71°, respectively. In the −55 kPa group, left and right knee ROM increased by 5.00° and 5.71°, respectively. Hip flexion ROM also improved after intervention. The groups with negative pressures from −45 kPa to −55 kPa had the greatest recovery. In the −35 kPa group, left and right hip flexion ROM increased by 4.29° and 6.43°, respectively. In the −45 kPa group, left and right hip flexion ROM increased by 6.43° and 4.29°, respectively. In the −55 kPa group, left and right hip flexion ROM increased by 5.72° and 8.57°, respectively.

In conclusion, NPCT with a negative pressure greater than −35 kPa exerts a stronger mechanical effect, which can restore muscle flexibility, reduce adhesions, and accelerate the recovery from exercise-induced fatigue.

A previous study on biomarkers of skeletal muscle micro-injury has demonstrated that changes in serum CK and LDH concentrations can effectively reflect alterations in sarcolemmal permeability and the extent of myofibril structural disruption (58). Exercise-induced mechanical stress activates calpain via the integrin focal adhesion kinase (FAK) signaling pathway, disrupting sarcolemmal integrity and causing CK (primarily the CK-MM isoenzyme) and LDH (characterized by an elevated LDH1/LDH2 ratio) to leak into the bloodstream (59). Consistent with the aforementioned research findings, participants in this study exhibited significant increases in serum CK and LDH levels 24 h after a single bout of high-intensity exercise (*P* < 0.01), confirming that high-intensity eccentric exercise can induce skeletal muscle micro-injury.

Analysis of serum CK and LDH levels in the control group and intervention groups (−25 kPa, −35 kPa, −45 kPa, and −55 kPa) at 24 h after exercise revealed that NPCT facilitated superior recovery of CK and LDH levels compared to the control group (*P* < 0.05), with the most effective recovery effect observed in the pressure range of −45 kPa to −55 kPa. In conclusion, NPCT can expedite the recovery from DOMS and reduce the time required for muscle injury repair.

Pain is associated with inflammation (60, 61). Pro-inflammatory cytokines TNF-α and IL-6 serve as key regulatory factors in the pathogenesis of DOMS, mediating inflammation-induced pain sensitization (62). TNF-α enhances nociception by activating the transient receptor potential vanilloid 1 (TRPV1) channel (63), while IL-6 promotes prostaglandin E2 (PGE2) synthesis by upregulating cyclooxygenase-2 (COX-2), establishing a positive feedback loop in neurogenic inflammation (64). Although more rapid clearance of TNF-α and IL-6 can alleviate post-injury pain (65), the molecular release mechanisms of these cytokines during DOMS remain unclear. In this study, serum IL-6 and TNF-α levels significantly increased 24 h after exercise (*P* < 0.01), indicating that the inflammatory response is consistent with the acute-phase response of muscle micro-injury. Participants who underwent cupping therapy exhibited lower levels of inflammatory markers post-exercise, although there was no statistically significant intergroup difference (*P* > 0.05). This aligns with the viewpoint of Ekrami et al. (66), who analyzed peripheral blood mononuclear cells of martial artists. This mechanism may be associated with Hsp27, which regulates inflammation (67). Exercise-induced mechanical stress upregulates Hsp transcription. Meanwhile, molecular chaperones inhibit NF-*κ*B signaling, blocking the transcription of IL-6 and TNF-α and thus sustaining muscle repair. The overexpression of Hsp27 exacerbates inflammation, and growing evidence highlights its role in inflammatory responses (68). It is worth noting that Pearson correlation analysis in this study revealed a significant positive relationship between elevated serum IL-6/TNF-α levels and Hsp27 expression (*r* > 0, *P* < 0.05), further supporting the association between post-exercise inflammation and Hsp27 activity. In summary, consistent with the findings of Ekrami et al. (69), our findings suggest that NPCT may reduce IL-6 and TNF-α levels via Hsp27 regulation (*r* > 0, *P* < 0.05), potentially suppressing the NF-κB signaling pathway and releasing anti-fatigue signaling factors to mitigate inflammatory responses.

In conclusion, based on the aforementioned analysis and discussion, NPCT demonstrates a positive effect in alleviating DOMS. The treatment mechanisms may be as follows: The suction pressure applied during cupping can facilitate the sustained stretching of locally damaged muscles, elevate skin temperature, improve muscle elasticity, reduce connective tissue adhesion, and diminish pain trigger points. Localized negative pressure can expedite blood circulation and metabolic turnover, ameliorate local hypoxia and ischemia. This aids in removing chemical byproducts, such as CK and LDH, from damaged tissues, thereby mitigating the extent of muscle damage. In addition to its hemodynamic effects, cupping also exhibits anti-inflammatory effects. Cupping post-exercise can augment localized blood flow, and localized negative pressure can induce capillary rupture, resulting in characteristic cupping marks. This process transforms physical stimuli into biological signals, thereby triggering inflammatory responses that promote tissue repair and adaptation. Collectively, these mechanisms suggest that NPCT not only addresses mechanical and metabolic aspects of DOMS but also engages immunomodulatory pathways to expedite the recovery process.

## 5 Conclusions

A single bout of high-intensity exercise induced DOMS in healthy male participants. NPCT demonstrated significant therapeutic efficacy. Specifically, among different pressure interventions of NPCT, those with higher pressures (ranging from −45 kPa to −55 kPa) were most effective in relieving exercise-induced muscle soreness. Moreover, these higher-pressure interventions could reduce DOMS-related symptoms, including swelling and stiffness, and accelerate the functional recovery of lower-limb explosive power and joint mobility. Future studies should refine pressure prescriptions (including ± 2.5 kPa gradient testing parameters and post-exercise timing windows). It should also verify sport-specific functional outcomes in athletes (including repeated jump kinetics or HIIT). Additionally, it should explore the gender-differential responses under hormonal control.

## 6 Limitations

The final sample size employed in this study was relatively small (*n* = 11 per group). This small sample size may limit the statistical power and generalizability of the research findings. Moreover, due to the absence of gender-stratified analysis, it is not feasible to draw conclusions regarding the potential gender-specific effects of NPCT on DOMS outcomes, considering the hormonal and biomechanical differences between genders. Future trials should recruit larger cohorts with balanced gender representation to explore the gender-dependent efficacy of NPCT. Personalized protocols should comprehensively take into account factors such as age, fitness status, and injury severity to align with the precision medicine framework. The data collection only encompassed the 24-hour time point post-exercise, overlooking the critical stages of DOMS progression. This oversight limits the understanding of the underlying mechanisms of the dynamic interaction between NPCT and inflammatory/metabolic recovery trajectories. Future research directions: (1) Performing multi-timepoint analyses (e.g., 0 h, 6 h, 24 h, 48 h, and 72 h) is essential for delineating the temporal dose-response relationships and optimizing the timing of intervention. The predominant reliance on quadriceps femoris studies inherently limits generalizability to muscles with distinct architectural configurations or functional specializations. (2) It is imperative to carry out comparative studies on diverse muscle groups (e.g., hamstrings, gastrocnemius, and deltoids) to determine muscle-specific therapeutic parameters. Mechanistic investigations should integrate biomechanical modeling and omics technologies to elucidate tissue-specific dose-response relationships.

## Data Availability

The original contributions presented in the study are included in the article/Supplementary Material, further inquiries can be directed to the corresponding authors.
